# Dynamic Visual Stimulations Produced in a Controlled Virtual Reality Environment Reveals Long-Lasting Postural Deficits in Children With Mild Traumatic Brain Injury

**DOI:** 10.3389/fneur.2021.596615

**Published:** 2021-11-25

**Authors:** Thomas Romeas, Selma Greffou, Remy Allard, Robert Forget, Michelle McKerral, Jocelyn Faubert, Isabelle Gagnon

**Affiliations:** ^1^Faubert Laboratory, École d'Optométrie, Université de Montréal, Montréal, QC, Canada; ^2^Institut National du Sport du Québec, Montréal, QC, Canada; ^3^School of Rehabilitation, Faculty of Medicine, Université de Montréal, Montréal, QC, Canada; ^4^Centre for Interdisciplinary Research in Rehabilitation of Greater Montreal - IURDPM, Montréal, QC, Canada; ^5^Department of Psychology, Université de Montréal and Centre for Interdisciplinary Research in Rehabilitation of Greater Montreal - IURDPM, Montréal, QC, Canada; ^6^Montreal Children's Hospital, McGill University Health Center, McGill University, Montréal, QC, Canada; ^7^School of Physical and Occupational Therapy, Faculty of Medicine and Health Sciences, McGill University, Montréal, QC, Canada

**Keywords:** mild traumatic brain injury (mTBI), postural instability, children, virtual reality, balance, sensorimotor control, perception-action coupling

## Abstract

Motor control deficits outlasting self-reported symptoms are often reported following mild traumatic brain injury (mTBI). The exact duration and nature of these deficits remains unknown. The current study aimed to compare postural responses to static or dynamic virtual visual inputs and during standard clinical tests of balance in 38 children between 9 and 18 years-of-age, at 2 weeks, 3 and 12 months post-concussion. Body sway amplitude (BSA) and postural instability (vRMS) were measured in a 3D virtual reality (VR) tunnel (i.e., optic flow) moving in the antero-posterior direction in different conditions. Measures derived from standard clinical balance evaluations (BOT-2, Timed tasks) and post-concussion symptoms (PCSS-R) were also assessed. Results were compared to those of 38 healthy non-injured children following a similar testing schedule and matched according to age, gender, and premorbid level of physical activity. Results highlighted greater postural response with BSA and vRMS measures at 3 months post-mTBI, but not at 12 months when compared to controls, whereas no differences were observed in post-concussion symptoms between mTBI and controls at 3 and 12 months. These deficits were specifically identified using measures of postural response in reaction to 3D dynamic visual inputs in the VR paradigm, while items from the BOT-2 and the 3 timed tasks did not reveal deficits at any of the test sessions. PCSS-R scores correlated between sessions and with the most challenging condition of the BOT-2 and as well as with the timed tasks, but not with BSA and vRMS. Scores obtained in the most challenging conditions of clinical balance tests also correlated weakly with BSA and vRMS measures in the dynamic conditions. These preliminary findings suggest that using 3D dynamic visual inputs such as optic flow in a controlled VR environment could help detect subtle postural impairments and inspire the development of clinical tools to guide rehabilitation and return to play recommendations.

## Introduction

Following mild traumatic brain injury (mTBI), postural problems are commonly reported in challenging situations (i.e., unstable support surface, absence of or conflicting visual information, added cognitive load) in both adults (1–8) and children (9, 10); yet, these findings are often ignored in clinical practice guidelines and not adequately tested before patients are discharged from follow-up programs.

Postural control in humans involves the integration of different sensory inputs to control motor output. These inputs are used both in a feed forward anticipatory mode to determine environmental context (e.g., body position and relation to gravity to plan movement) and in a feedback mode to correct posture. Some have suggested that deficits in the visual system contribute significantly to postural instability following mTBI (2, 11). Indeed, residual sensory integration dysfunction can be seen 30 days post-injury, manifesting as postural deficits induced by visual field motion (5). Postural instability is the inability to maintain equilibrium under dynamic (i.e., perturbations) and static (i.e., quiet stance) conditions. In the pediatric population, slow response time on a visuo-motor task in children with mTBI vs. control subjects 3 months post-injury (12) has been reported. Moreover, a study by our group using psychophysical assessment tools has shown poor integration of higher level visual information (i.e., second order stimuli requiring higher-level visual cortical function), which persisted up to 3 months in children aged 8–16 years following mTBI (13). The crucial role of vision in postural control in children has been shown using a swinging room paradigm that induced visual flow (14) or using fully immersive 3D dynamic virtual reality (VR) stimuli (i.e., Virtual Tunnel Paradigm) (15, 16). The 3D perspective control created in VR allows the design of more ecological (i.e., real-life) and challenging visual environments (17), while also maintaining safety and providing controlled conditions for the researcher. For example, the perception of visual motion in VR has shown strong destabilizing effects on standing posture in typically developing children up to 16 years of age (18). To our knowledge, no previous study has investigated the role of high-level visuo-motor integration in postural control in children following mTBI while measuring the evolution of such a behavior prospectively post-injury. In addition, previous studies have emphasized the limitations associated with clinical balance tests (i.e., Balance Error Scoring System [BESS]) in the assessment of postural deficits after mTBI, including insufficient repeatability, poor reliability, fatigue effects, influences from musculoskeletal injuries and learning effects (19–24). A number of recent reviews and meta-analyses have also pointed out potential persistent motor system and attentional deficits following mTBI that are not detected with standard clinical balance tests, but that would lead to an increase risk of neuromuscular injuries (i.e., lower extremity injury) within the year following an mTBI (25–27).

The objectives of the current study were to investigate the impact of mTBI in children on postural responses using the Virtual Tunnel Paradigm as well as clinical measures of balance (Bruininks-Oseretsky Test of Motor Proficiency, Second Edition [BOT-2] and timed tasks) at 2 weeks, 3 months, and 12 months post-injury. In addition, we also aimed to examine the relationship between self-reported post-concussion symptoms and postural stability performance in children with mTBI.

## Methods

### Participants

Seventy-six children between 9 and 18 years of age participated in the study (Table 1). Thirty-eight children with mTBI [13 females; mean age ± SD (years) at 2 weeks (i.e., first session): 14.08 ± 2.52; 3 months: 14.29 ± 2.53; 12 months: 15.04 ± 2.52] were recruited from the Trauma Center of The Montreal Children's Hospital if they had: (1) a diagnosis of mTBI as defined by the WHO task force (28); (2) no premorbid medical diagnosis of learning disabilities, attention-deficit/hyperactivity disorder, postural problems (e.g., vestibular disorder), and/or behavioral problems; (3) no comorbid orthopedic or musculoskeletal injury, and (4) no previous mTBI in the last 6 months (other than the index injury) or persisting symptoms from a previous mTBI. In addition, 38 healthy control participants [13 females; mean age± SD (years) at first session: 14.02 ± 2.66; 3 months: 14.25 ± 2.66; 12 months: 15.02 ± 2.66] were recruited among the friends of mTBI participants (as an attempt to match participants' socio-economic status). They were matched according to age, gender, and premorbid level of physical activity assessed with the Activity Rating Scale (29) which is a single question self-report of general activity levels used in the context of epidemiological studies, and related to general fitness in individuals. The level of participation in their specific sport was also quantified in terms of the number of training sessions per week (mTBI: 3.08 ± 2.10; Controls: 2.63 ± 1.62), number of hours of training per week (mTBI: 5.25 ± 4.19; Controls: 3.99 ± 2.42) and years of practice (mTBI: 5.32 ± 4.22; Controls: 5.69 ± 3.86). A previous diagnosis of mTBI was considered as an exclusion criterion for control participants and the same exclusion criteria (2 and 3) as for the mTBI group were applied. The study received the approval from the institutional research ethics boards of the Université de Montréal and of the McGill University Health Center.

**Table 1:** Characteristics of mTBI participants.

**mTBI characteristics**	**Number**	**Percentage**
**Gender**		
Male	25	66
Female	13	34
**Cause of injury**		
Falls	7	18
Hits (direct hit to the head or body; e.g., punch, ball)	25	66
Hits followed by a fall	5	13
MVA-bicycle	1	3
**Injury associated with sport**		
Sport associated	32	84
Non-sport associated	6	16
**Admission GCS score**		
13	0	0
14	1	3
15	24	63
Unknown	13	34
**Duration of LOC**		
No LOC	22	58
0–1 min	5	13
>1min	1	3
Unknown	10	26
**Duration of PTA**		
0-60 min	5	13
>60 min	0	0
Unknown	33	87
**Concussion grade**		
Trivial	0	0
Simple	0	0
Complex	1	3
Unknown	37	97
**Symptoms at the time of injury**		
Headache	32	84
Nausea-vomiting	15	39
Dizziness	27	71
Visual problems	6	16
Drowsiness	6	16
Sonophobia	4	11
Photophobia	8	21
Difficulty concentrating	5	13
Fatigue	9	24
**Session characteristics**	**Mean**	**SD**
Age at first session (years)	14.08	2.52
Days after mTBI (first session)	17.00	4.84
Age at second session (years)	14.29	2.53
Days after mTBI (second session)	93.07	15.65
Age at third session (years)	15.04	2.52
Days after mTBI (third session)	368.26	36.58

*MVA, motor vehicle accident; GCS, Glasgow Coma Scale; LOC, loss of consciousness; PTA, post traumatic amnesia.

### General Procedures

All participants had normal or corrected-to-normal vision as confirmed by a certified optometrist on the day of the test. During the session, they filled in the Post-Concussion Symptom Scale-Revised (PCSS-R) (30), and underwent postural testing. This protocol was performed 2 weeks (mean days ± SD: 17.00 ± 4.84), 3 months (93.07 ± 15.65), and 12 months (368.26 ± 36.58) post-injury and about 2.5 months (83.28 ± 19.03), and 11.5 months (364.23 ± 14.85) after the first visit of control participants in order to be consistent with the in-between session duration in both groups.

### Postural Assessment Tools

#### VR Postural Test

**Figure 1:**
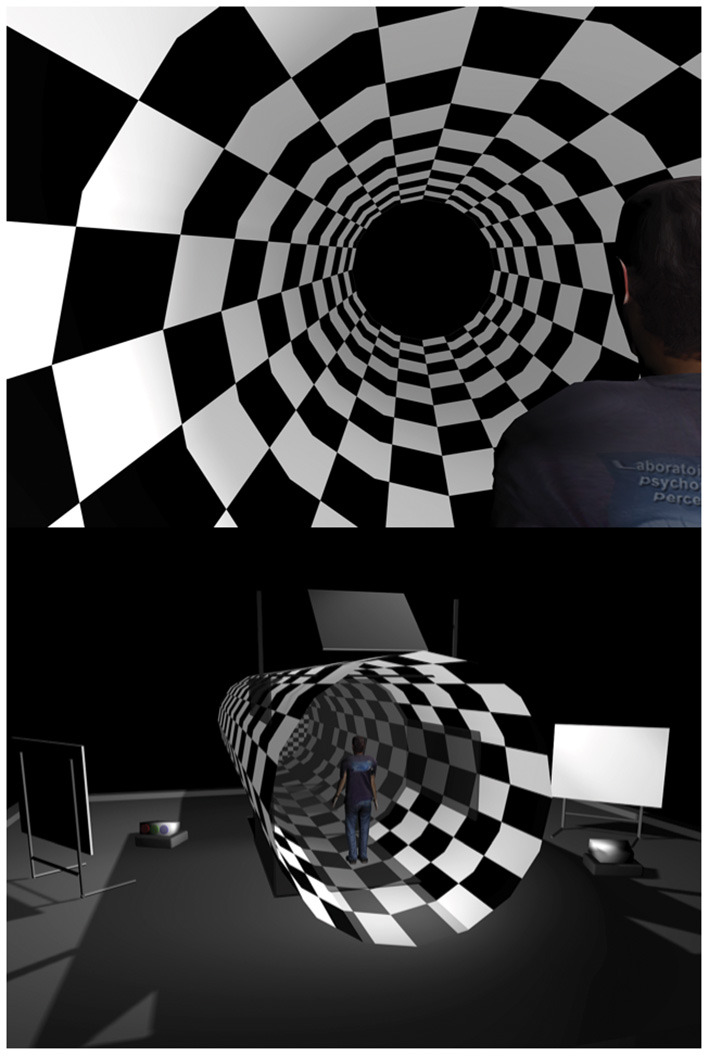
The Virtual Tunnel Paradigm.

The Virtual Tunnel Paradigm was used to induce visual disturbances during quiet stance (15, 16, 18). It consisted of a virtual tunnel whose inner texture was akin to a checkerboard pattern, where each square was scaled for linear perspective (Figure 1). The white squares had a luminance of 47 cd/m^2^ and the black squares 0.52 cd/m^2^ (98% Michelson contrast). The tunnel's virtual length was 20 m and its diameter 3 m; these dimensions remained constant across all trials. The tunnel was produced by the projectors of a fully immersive virtual environment (the CAVE system; Fakespace™). In this study, two visual disturbance conditions were used: *Dynamic* and *Static Tunnel Conditions*. In the *Dynamic Tunnel Condition*, the virtual tunnel moved in an anterior–posterior direction obeying a sinusoidal translation function with a peak-to-peak amplitude of 2 m and oscillating around the participants at three different translation frequencies of 0.125, 0.25, or 0.5 Hz. In the *Static Tunnel Condition*, the tunnel remained stationary (0 Hz, amplitude of 0), resulting in a total of 4 trials presented in a random order. Each trial lasted 68 s, but in order to eliminate transitory postural responses that occur during the first few seconds after the stimulus appears, the analysis was limited to the last 64 s of stimulus presentation. Therefore, a total of four 64-s trials recorded at a rate of 40 Hz by the motion tracker system (Flock-of-Birds, Ascension Technology Corporation) were analyzed: one at each of the three stimulation frequencies in the dynamic condition and one at the 0 Hz frequency in the static condition. During the test, participants were asked to wear the stereoscopic goggles (CrystalEyes^TM^) alternating at a frequency of 96 Hz, which allowed them to perceive the 3D characteristic of the environment, but also allowed to record changes in posture using a magnetic motion tracker system that registers the movement of an individual's head with magnetic motion sensors. This system also allowed to record the height of mTBI [mean ± SD (m) at 2 weeks: 1.44 ± 0.15; 3 months: 1.45 ± 0.15; 12 months: 1.48 ± 0.12] and Controls (2 weeks: 1.42 ± 0.15; 3 months: 1.44 ± 0.15; 12 months: 1.47 ± 0.13) at the eye level, which was estimated at the time 0 of each trial based on the Y position (elevation) of the tracker. Participants were then positioned 1.50 m from the CAVE's central wall without shoes, feet together, and arms crossed. This position was chosen to minimize the use of individual strategies from the limbs to maintain posture and help maximize the effect of the stimulation. For all conditions, they were asked to fixate a red dot located at the horizon.

**Figure 2:**
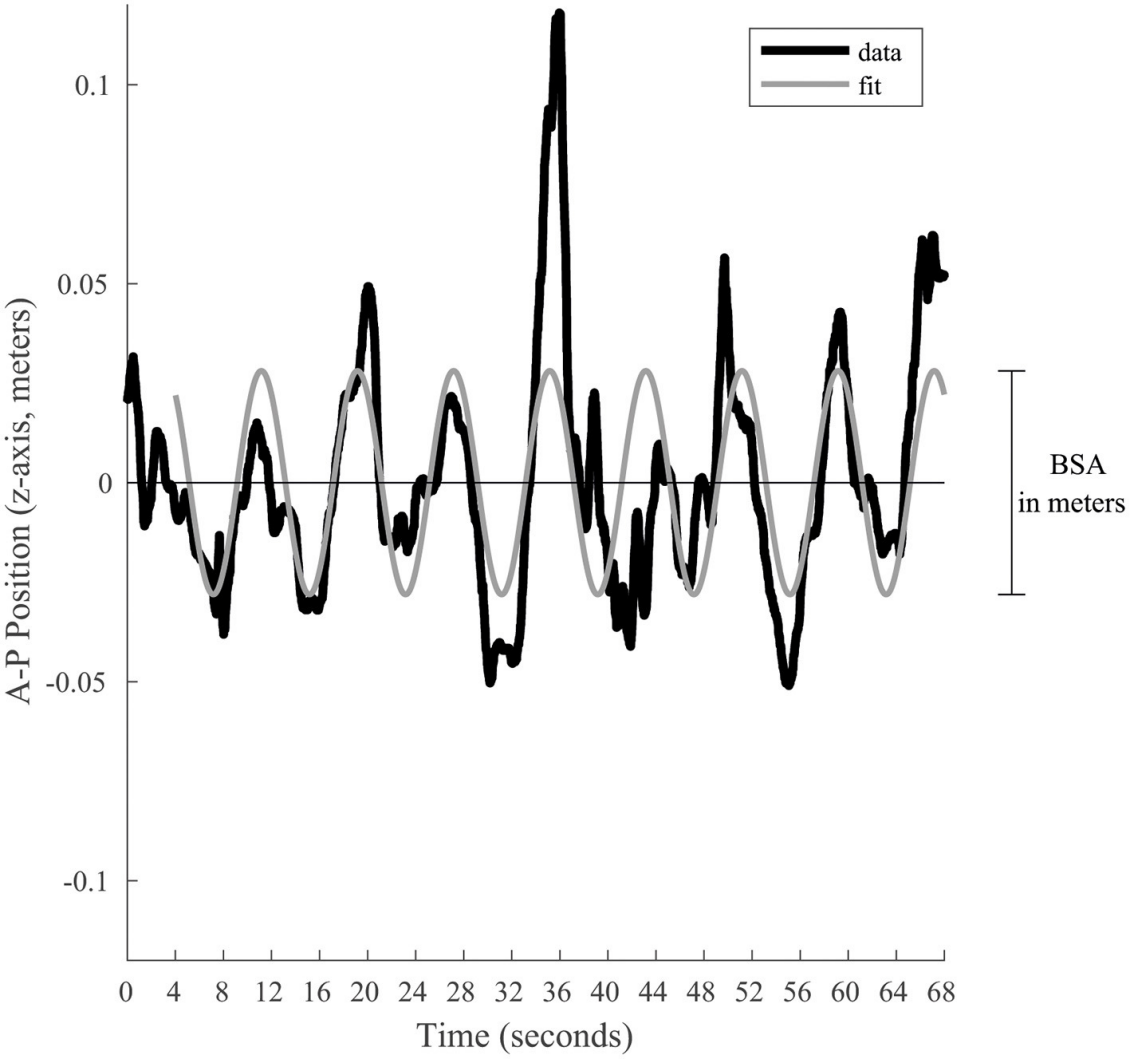
Graphical representation of the BSA calculation for a given trial. The black line represents the position along the anterior-posterior axis. To estimate the postural response of the subject, a sine wave (gray line) is fitted to the last 64 s. This sine wave is constrained to have a frequency equal to the visual stimulation. Its total amplitude represents the BSA in meters, which is afterwards converted in degrees of angular displacement.

Two main postural stability indicators were extrapolated: *Body Sway Amplitude* and *Postural Instability* (16). *Body Sway Amplitude* (*BSA*) reflects the participant's reaction to, and synchronization with, a given visual stimulus (5, 16, 18, 31–36). It corresponds to the average anterior–posterior (AP, which corresponded to the Z position of the tracker) displacement of a participant measured for one given oscillation frequency, during the last 64 s of a trial at a given stimulus oscillation frequency. The BSA was calculated by fitting a sine wave to the Z position data (i.e., position along the anterior-posterior axis, Figure 2) that has the same temporal frequency as the signal, which minimizes the root mean square error. The BSA was defined as the total amplitude (difference between peak and trough of the fitted sine wave) of the sine wave. The fit of the sine wave was computed according to the tracker position (in meters). To take into account the subject's height, the BSA was converted into the angle of motion of the tracker relative to the subject's feet [*BSA*_*degrees*_ = 2 × atan ((*BSA*_*meters*_/2)/*h*), where *h* is the height of the head tracker]. *Postural Instability* (*vRMS*) quantified the subject's instability based on the X, Y, and Z position data from the tracking system. Because instability along the elevation axis should be small, the data from the Y channel were not considered; only the position along the medial-lateral and AP axes (data from the X and Z channels, respectively) were considered. To minimize the position artifacts related to the tracker recording, the data from each of the X and Z channels were filtered in the Fourier domain to remove all frequencies above 8 Hz (i.e., we applied a fast Fourier transform to each channel and then filtered each channel with a low-pass filter to eliminate signal above the cut-off frequency of 8 Hz). In other words, temporal frequencies above 8 Hz were attributed to tracker noise and were discarded in the postural instability. Furthermore, because the postural sway with the stimulus is not considered instability, the postural response at the temporal frequency of the stimulation along the AP displacement that is responsible for the BSA was also filtered out in the Fourier domain (i.e., a notch filter was used to remove the stimulus frequency in only the X channel). In sum, the X channel (AP axis) was low-pass filtered (<8 Hz) and notch filtered to remove stimulus frequency, the Z channel (medial-lateral axis) was only low-pass filtered (<8 Hz), and the Y channel was discarded. After filtering the position data, the data from the X and Z channels in the Fourier domain were converted back to the time domain (inverse fast Fourier transform) resulting into a 2D position vector for each time point. Then, this 2D position data as a function of time was numerically differentiated to obtain absolute velocity as a function of time (i.e., vector length of the derivative of the 2D position data). The *vRMS* was calculated as the root mean square (rms) of the absolute velocities as a function of time. Analogously to the BSA, the units were converted from m/s to degrees/s to consider the height of the subject. Thus, the postural instability was derived by calculating the root mean squared (vRMS) of total body velocity (recorded at the head level) in the anterior–posterior and medial-lateral planes in degrees of angle per second. vRMS here is similar to a previously used Instability Index (33, 36), expect for the filtering out of some information.

#### Clinical Balance Tests

For this study, three items from the balance subtest of the BOT-2 were used. Previous work by our group, using the BOT 1^st^ edition in a similar population had shown that some individual items were more sensitive to the mTBI than others (9). With the second edition of the BOT, some of the items used in our previous work were no longer available, so we chose the 3 most challenging items from the BOT-2 to examine more specifically in the current study. Using individual items requires the use of raw scores, but with groups matched for age and sex, raw scores could be compared across groups. *Item 3*: standing single leg on a line with eyes open; *Item 6*: on a line with eyes closed; *Item 9*: on a beam (narrow surface) with eyes closed. We also administered three timed tasks, where participants were standing on a foam surface, eyes closed, with hands on hips, in various base of support conditions. We recorded the number of seconds without deviating from the prescribed position (e.g., Hands lifted off iliac crest, Opening eyes, Step, stumble, or fall) in: *Task 1*: double-leg stance with feet together; *Task 2*: single-leg stance; Task *3*: tandem stance. The three timed tasks were derived from the Pediatric Clinical Test of Sensory Interaction for Balance, which we had previously used in our work (9, 37), but also by others in the context of other populations [e.g., (38)] Both clinical tests were administered by three trained evaluators, two of whom are co-authors on this paper (SG, TR).

### Statistical Analyses

#### VR Postural Test

A log transformation was used for both the BSA (degree of angular displacement) and the vRMS (degree/s) to conform to normality of distribution. However, note that in the figures presented in the article, we used linear values in order to facilitate interpretation. Factorial repeated linear mixed models (first-order heterogeneous factor-analytic covariance structure) were used for vRMS and BSA to probe between-subjects *Group* differences (mTBI vs. Controls) and across within-subject factors, namely *Time* representing time and test sessions post-injury (2 weeks, 3 months, and 12 months), *Condition* (Dynamic vs. Static tunnel), and *Frequency* of tunnel oscillation (0.125, 0.25, and 0.50 Hz). *Age* was used as a categorical covariate (above and below 16 years) because of the previously demonstrated effect of age on visually-driven postural behavior (vRMS) using the Virtual Tunnel Paradigm (18). Pairwise comparisons were made using Sidak corrections.

#### Clinical Balance Tests

A mixed design repeated measures analysis of covariance (ANCOVA) was used to probe between-subjects *Group* differences across within-subject factors, namely *Time* and *Items* of the BOT-2 (3, 6, and 9) and of the three timed tasks (1, 2, and 3); *Age* categories were used as a covariate.

#### Symptoms

A mixed design repeated measures ANOVA was used to probe between-subjects *Group* differences across the within-subject factor *Time* on the total score of symptoms (PCSS-R). Spearman rank correlation coefficient was used to investigate the possible associations between PCSS-R and the different measures of postural response.

## Results

### VR Postural Test

#### BSA

**Figure 3:**
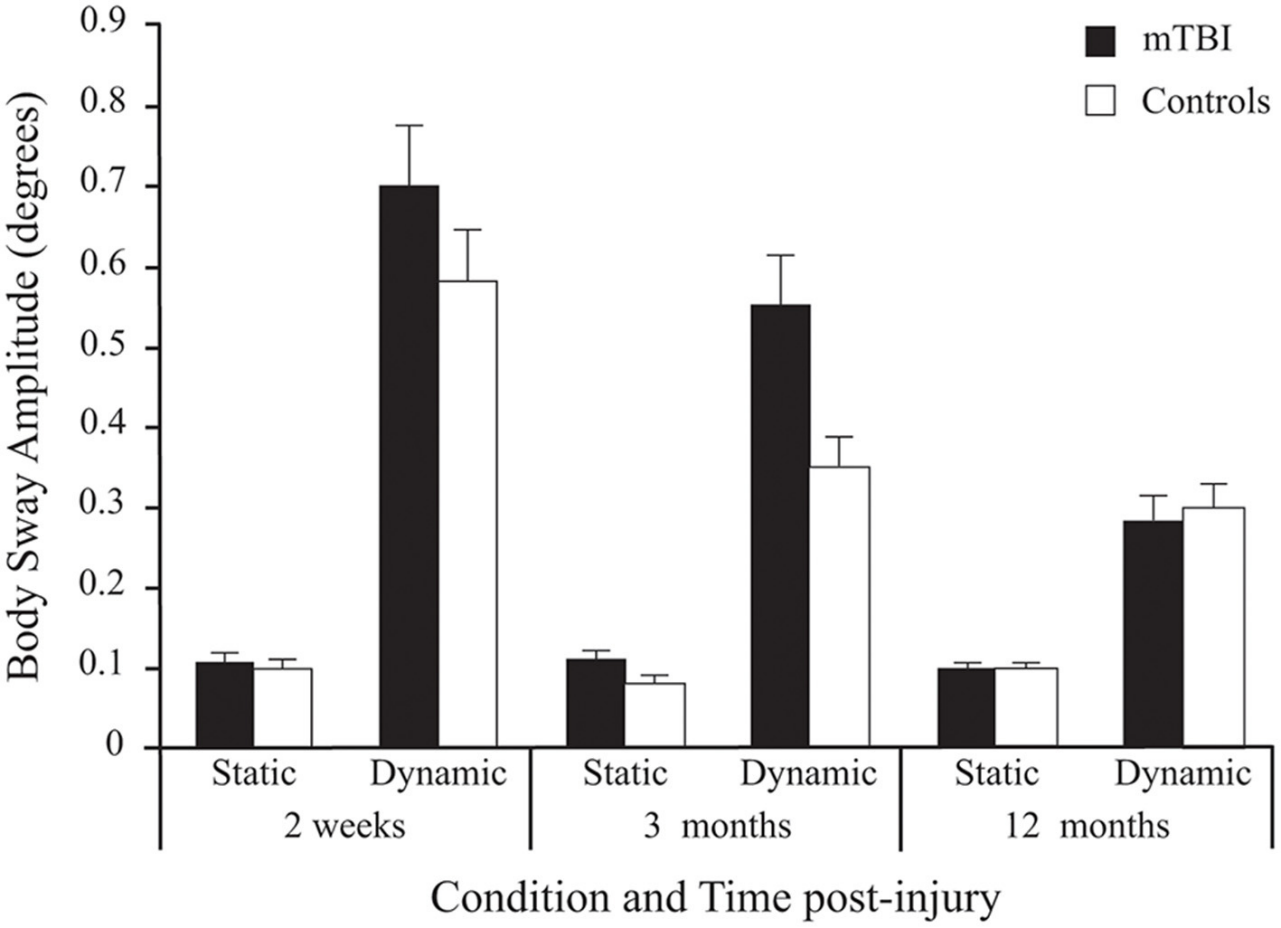
Between-Group differences (Mean ± SEM mTBI vs. Mean ± SEM Controls) in Body Sway Amplitude (degrees) as a function of Condition and Time post-injury (Session). Note that since stimulus Frequency did not significantly influence inter-group differences, only average values of the three frequencies are shown here.

The mTBI participants swayed significantly more than the Control participants as evidenced by a strong significant *Group* effect (see Table 2 for statistical values of main effects). As can be seen in Figure 3, results also showed that all participants' postural response in phase with the stimulus (BSA) increased significantly more when the Virtual Tunnel Paradigm was Dynamic compared to when it was Static. A main effect of *Age* was shown, with younger participants exhibiting more postural response than older ones.

**Table 2:** Statistical values of significant results in all postural assessments.

**Postural assessment**	**Source**	**Num. DOF**	**Den. DOF**	***F***	**Sign**.
vRMS	Group	1	185	63.29	*p* < 0.001
	Condition	1	185	123.22	*p* < 0.001
	Time (i.e., Sessions)	2	154	5.67	*p* = 0.004
	Age categories	1	179	117.04	*p* < 0.001
	Group × Condition	1	185	5.38	*p* = 0.021
	Group × Time	2	154	5.51	*p* = 0.005
	Condition × Time	2	154	4.42	*p*= 0.014
	Condition × Age	1	179	35.03	*p* < 0.001
	Group × Condition × Time	2	154	3.69	*p* = 0.027
BSA	Group	1	71	21.96	*p* < 0.001
	Condition	1	71	413.78	*p* < 0.001
	Age categories	1	69	45.72	*p* < 0.001
	Group × condition	1	71	7.78	*p* = 0.007
	Group × Time	2	95	8.65	*p* < 0.001
	Group × Condition × Time	2	95	4.14	*p* = 0.019
	Frequency	2	108	26.8	*p* < 0.001
BOT-2	Items	2	144	42.09	*p* < 0.001
Timed tasks	Time	2	142	8.9	*p* < 0.001
	Time × Age	2	142	3.578	*p* < 0.035
	Items	2	142	120.51	*p* < 0.001

*vRMS, velocity root mean square; BSA, body sway amplitude; Num. DOF, numerator's degree of freedom; Den. DOF, denominator's degree of freedom; F, F-value; Sign, degree of significance*.

Although there was no significant main effect for *Time*, there was a *Group* × *Time* interaction. Pairwise comparisons revealed that BSA difference between groups was significant at 2 weeks (*p* < 0.029) and 3 months (*p* < 0.001), but not at 12 months post-injury (*p* = 0.940).

A significant *Group* × *Condition* × *Time* interaction was also found. In both groups, there was no effect of *Time* in the Static condition (*p* = 0.548), but a decrease in BSA (i.e., lower response) in the Dynamic condition (*p* < 0.001). In the mTBI group, the decrease only showed a tendency between 2 weeks and 3 months (*p* = 0.08), but was significant between 3 and 12 months (*p* < 0.001) and between 2 weeks and 12 months (*p* < 0.001). Note that in the Control group, a significant decrease between 2 weeks and 12 months (*p* < 0.001) was mostly produced by a decrease between 2 weeks and 3 months (*p* < 0.006) since there was no significant difference between 3 and 12 months (*p* = 0.115).

There was a significant main effect of *Frequency* for BSA, with the lowest frequencies (0.125 and 0.25 Hz) inducing the most postural response when compared to the fastest one (0.50 Hz) at each *Time* and in each *group*.

#### vRMS

**Figure 4:**
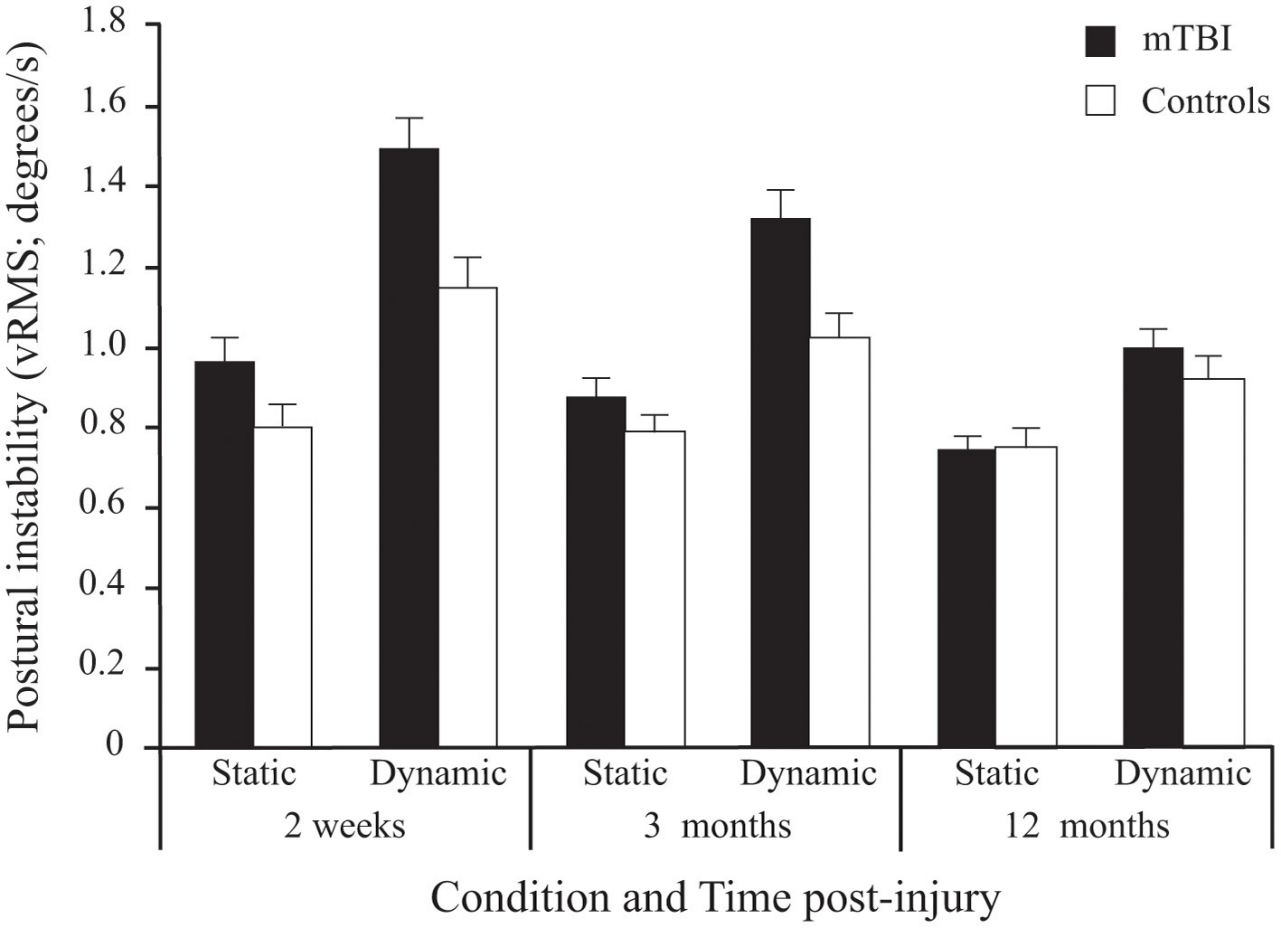
Between-Group differences (Mean ± SEM mTBI vs. Mean ± SEM Controls) in Postural Instability (vRMS; degrees/s) as a function of Condition and Time post-injury (Session). Note that since stimulus Frequency did not significantly influence inter-group differences, only average values of the three frequencies are shown here.

A significant *Group* effect confirmed that mTBI participants had more postural instability than Control participants (see Table 2). Expectedly, all participants demonstrated more postural instability when the Virtual Tunnel Paradigm was dynamic compared to when it was static (i.e., *Condition* effect) and the difference between groups was greater in the dynamic condition compared to the static condition (*Group x Condition* interaction; Figure 4). There was a main effect of *Age*, with younger participants exhibiting more instability than older ones and a *Condition* × *Age* interaction showing this to be more evident in the dynamic condition. There was also a *Time* main effect where vRMS decreased (i.e., lower instability) with time and a significant *Group* × *Time* interaction showing that this decrease was more pronounced in the mTBI group. Pairwise comparisons revealed that body vRMS difference between groups was significant at 2 weeks (*p* < 0.001) and 3 months (*p* < 0.001), but not at 12 months post-injury (*p* = 0.449). A significant *Group* × *Condition* × *Time* interaction was also found. In the mTBI group, for the Static condition, vRMS was similar at 2 weeks and 3 months (*p* = 0.797), showed a tendency to decrease between 3 and 12 months (*p* = 0.085) and was different between 2 weeks and 12 months (*p* < 0.017). In the Dynamic condition, there was also no significant difference between 2 weeks and 3 months (*p* = 0.182), but the decrease was significant between 3 and 12 months (*p* = 0.001) and between 2 weeks and 12 months (*p* < 0.001). In the Control group, there was no significant effect of *Time* for the Static condition (*p* = 0.442), but a significant effect in the Dynamic condition (*p* < 0.001), where vRMS decreased slightly between 2 weeks and 12 months (*p* < 0.022). Finally, results showed no main effect of Frequency and no interaction implicating the *Frequency* factor.

### Clinical Balance Tests

#### BOT-2

Contrary to what was found on the visually-induced postural control measures (Virtual Tunnel Paradigm), results showed no significant main effect of *Group* or *Time* on the administered BOT-2 items. However, a significant main effect of *Items* across *Groups* and *Time* was found (Table 2). More specifically, Sidak pairwise comparisons revealed that Item 9 was more difficult (lower hold-time values) than Item 6, which in turn was more complex than Item 3 (*p* < 0.001).

#### Timed Balance Tasks

**Figure 5:**
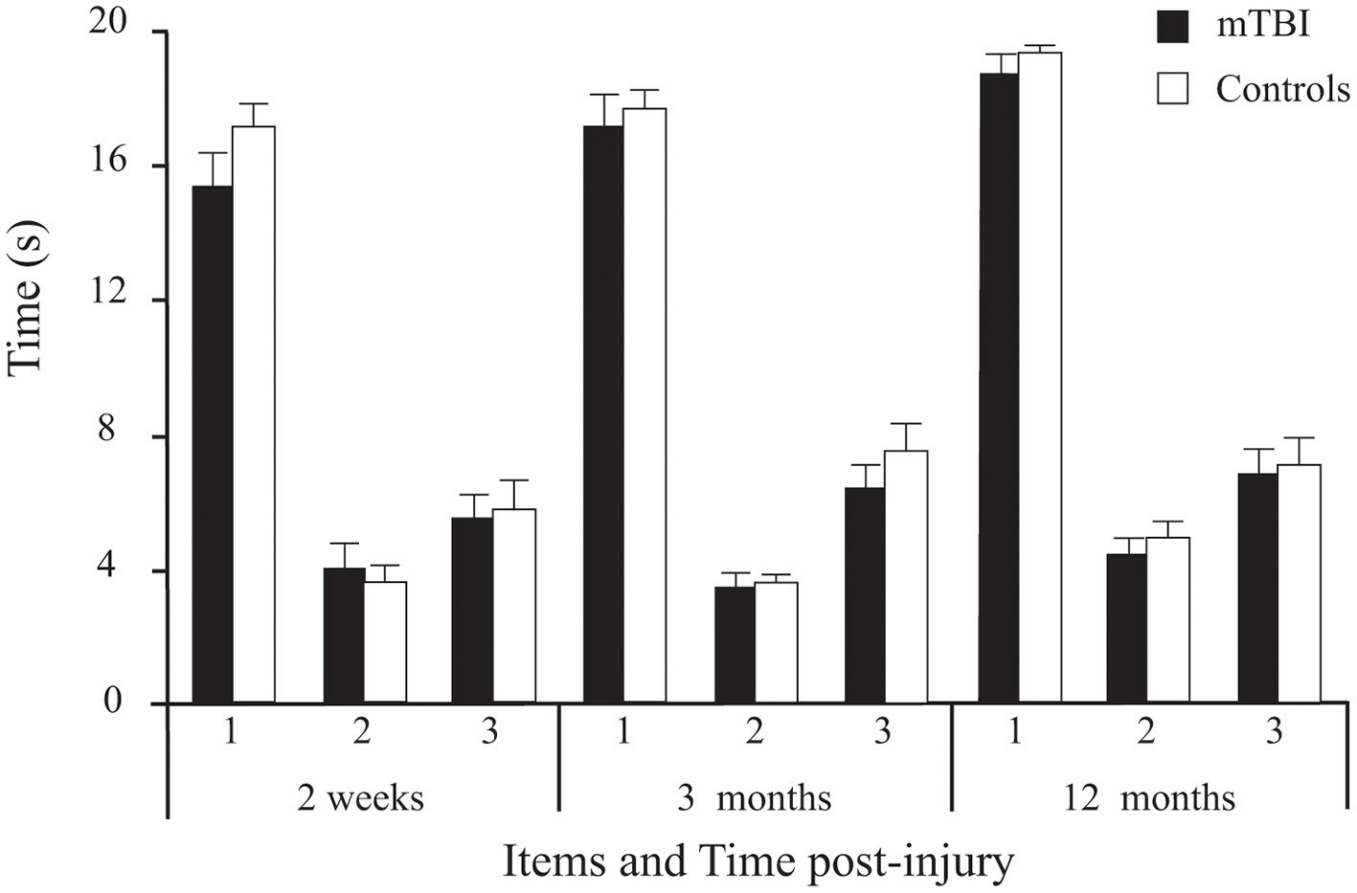
Between-Group differences (Mean ± SEM mTBI vs. Mean ± SEM Controls) on the three timed tasks (average duration of correct stance of the 2 trials/ item, in seconds) as a function of Time post-injury (Session).

Results showed no significant main effect of *Group* on the 3 timed tasks. However, a significant main effect of *Time* was found (Table 2; Figure 5), with Sidak comparisons showing performances improving slightly (higher holding-time) at 3 months compared to 2 weeks (*p* = 0.039) and improving further at 12 months compared to 3 months (*p* = 0.007). There was also a significant *Time* × *Age* interaction showing that this balance improvement with time was linked to younger individuals. There was a significant *Item* main effect. More specifically, single leg stance (Item 2) was more difficult (lower time values) than tandem stance (Item 3), which in turn was more difficult than double leg stance (Item 1; *p* < 0.001) in both groups.

#### Correlations

Clinical measures of balance showed moderate to weak negative correlations with BSA and vRMS in the virtual room, indicating that less time spent in a position with the clinical tasks correlated with more postural instability. Among the six different balance tasks of the clinical tests, the two easiest tasks, that is Item 3 of BOT-2 (standing on one leg on floor with eyes opened) and the first timed task (standing feet together on foam with eyes closed), did not show correlations with BSA or vRMS in either group. For the mTBI group, strongest correlations were found between Item 6 of BOT-2 (standing on one leg on the floor with eyes closed) and vRMS (*r*_*s*_ = −0.537, *p* < 0.001) as well as BSA (*r*_*s*_ = −0.476, *p* = 0.003), in the dynamic tunnel conditions. For the control group, significant, but weaker, correlations were found between the second timed task (standing in tandem on the foam with eyes closed) and vRMS (*r*_*s*_ = −0.424, *p* = 0.013) as well as with BSA (*r*_*s*_ = −0.458, *p* = 0.007), also in the dynamic tunnel conditions. None of the clinical tasks correlated with BSA in static conditions in either group.

### Post-concussion Symptoms

#### PCSS-R Total Score

**Figure 6:**
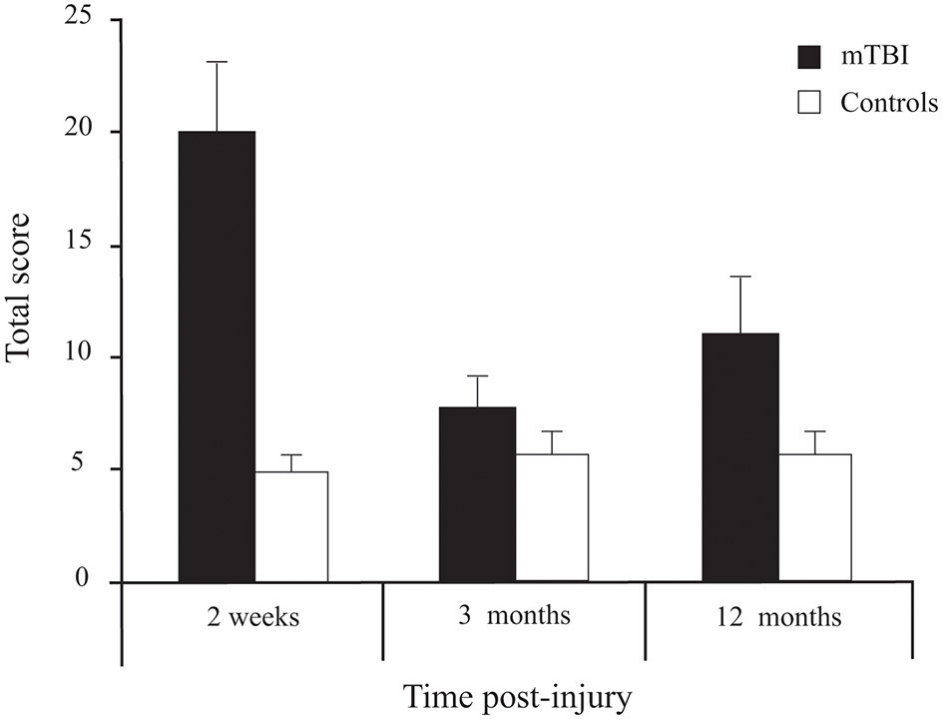
Between-Group differences (Mean ± SEM mTBI vs. Mean ± SEM Controls) for Total Score on the Post-Concussion Symptom Scale—Revised as a function of Time post-injury (Session).

Participants with mTBI had higher scores than controls [*Group* effect: *F*_(1, 72)_ = 11.143, *p* = 0.001] (Figure 6). Moreover, a *Group* × *Time* interaction [*F*_(2, 144)_ = 12.793, *p* < 0.001] confirmed that the symptoms decreased with time in the mTBI participants only. Sidak pairwise comparisons revealed that PCSS-R scores in participants with mTBI were highest at 2 weeks compared to 3 months (*p* < 0.001) and 12 months post-injury (*p* = 0.005), but no significant difference existed between 3 and 12 months (*p* = 0.506). Unpaired Student's *t*-tests showed that PCSS-R score differences between groups were significant only at 2 weeks post-injury [*t*_(74)_ = 4.746, *p* < 0.001)], but not at 3 months [*t*_(73)_ = 1.272, *p* = 0.207] nor at 12 months post-injury [*t*_(74)_ = 1.872, *p* = 0.065].

#### Correlations

Significant correlations were found between total PCSS-R score at 2 weeks post-trauma and item 6 of BOT-2 (standing on one leg on the floor with eyes closed) at 2 weeks post-trauma (*r*_*s*_ = −0.422, *p* = 0.008) and the 3^rd^ timed task (tandem on a foam eyes closed) at 12 months post-trauma (*r*_*s*_ = −0.441, *p* = 0.006). In these two cases, fewer symptoms were associated with higher balance scores. No significant correlations were found between symptoms and BSA or vRMS in the virtual tunnel.

## Discussion

Results revealed that despite no difference in self-reported symptoms at 3 and 12 months between mTBI and control groups, postural deficits were still present 3 months post-mTBI, but had disappeared by 12 months in this cohort of children. Moreover, these deficits were best found when postural response was tested in reaction to dynamic compared to static visual inputs in a VR environment (Virtual Tunnel Paradigm). On the other hand, standard clinical balance evaluations such as the BOT-2 and timed tasks did not reveal postural deficits within 2 weeks, 3, and 12 months post-injury.

### Impaired Postural Control After mTBI

Earlier studies had shown that children with mTBI had lower motor performance in the domains of postural stability and response speed (10) and that postural deficits were still present at 3 months post-injury despite rapid reduction of self-reported symptoms (9). One of the most important findings in the current study was that the postural anomalies found with the instrumented measures of postural response (i.e., BSA and vRMS) in the VR paradigm were no longer present by 12 months post-injury, suggesting that the mTBI cohort recovered their postural stability between 3 and 12 months. As we will discuss later in the clinical implications section, this finding could have important impacts in guiding more effective rehabilitation and criteria for return to play.

### Dynamic vs. Static Environments

Children with mTBI showed greater body sway amplitude (BSA) and postural instability (vRMS) compared to their uninjured peers at 2 weeks and 3 months after the injury when exposed to complex dynamic visual environments (dynamic Virtual Tunnel Paradigm), but less so under less challenging conditions (static Virtual Tunnel Paradigm). The difference between dynamic vs. static conditions suggests that the added complexity of the sensory information to be processed and integrated by the postural control system might be the key element to this difference. Interestingly, studies that have shown postural difficulties following concussion or mTBI in adults have proposed a central sensory integration difficulties hypothesis to explain their findings rather than the presence of motor deficits *per se*. In this previous work, the most challenging situations, i.e., unstable support surface, absence or conflicting visual information (2–5, 9), and added cognitive load (6, 7, 27, 39–42) induced the most postural difficulties. In line with the relevance of using more challenging dynamic stimulation, VR technology has recently been proposed as a useful tool for postural assessment, rehabilitation and to detect subacute mTBI deficits (43–48). For example, a recent study demonstrated that specific postural tasks designed to assess visual-vestibular inputs in dynamic immersive VR environment were found to be the most sensitive tests for discriminating health status following mTBI while the BESS, King-Devick and Dynamic Visual Acuity tests did not detect any differences between mTBI and control groups (49). A pilot study also suggested that VR based-therapy could help TBI patients improve their dynamic and static postural stability as well as gait and arm movements (50). Our study aligns with this evidence, showing that subtle postural deficits are still present 3 months post-mTBI when children are assessed with virtual dynamic inputs.

### Deficits in Visual Integration After mTBI

The dynamic aspect of the tasks brought by optic flow in the Virtual Tunnel Paradigm challenged postural control. Previous work has suggested that the visual system may play a key role in the production of postural difficulties following mTBI. In a study performed on adults, it was suggested that sustaining mTBI induces an over-reliance on visual input when regulating posture a few days post-injury (11). Another study has shown that college athletes with mTBI failed to appropriately use visual cues to regulate their posture when assessed using the Sensory Organization Test (2). Yet another study on concussed teenage athletes (18 years old) showed that after mTBI, there was decreased stability up to ~3 days after the injury, which appeared to be related to a sensory integration problem, whereby the injured athletes failed to use their visual system effectively (51). Moreover, a study by our group has shown that children aged 8–16 years with mTBI presented selective processing deficits for higher-order visual information (complex 2^nd^ order stimuli) and that this deficit was still present 3 months after injury (13). Likewise, selective deficits in complex visual information processing were demonstrated in adults with mTBI as evidenced by abnormal visual evoked potentials in response to complex visual stimuli (52, 53). In a study by our group (54), adults with mTBI showed longer correct-response reaction time means to sinewave gratings (i.e., patterns of bars from varying light intensity) compared to their healthy peers at 2 weeks, 3, and 12 months; hence suggesting persistence of mTBI-induced visuomotor anomalies.

### Standard Clinical Tests of Balance Control

In the present study, between-group differences were not significant on clinical tests of balance. This contradicts previous findings where children with mTBI, aged 7–16 years old, showed postural deficits 3 months post-injury on the Balance subtest of the BOT 1^st^ edition and on the eyes closed condition of the Pediatric Clinical Test of Sensory Interaction for Balance (9). Gagnon et al. (9) studied younger children and used the BOT 1^st^ edition which included tandem walking and obstacle crossing, more complex tasks involving movement and processing of dynamic visual inputs, which are not included in the BOT-2.

The fact that participants with mTBI significantly differed from their uninjured controls in their postural behavior (more so in vRMS than in BSA) on the Static tunnel condition (2 feet together, staring at the horizon with eyes open) and not on the BOT-2 Item 3 (standing only on the dominant leg on a line with eyes open and staring at a fixed point) is surprising, as the latter test is more challenging. This difference could be explained by the fact that vRMS and BSA are precise measures with a high sampling rate (40 Hz) and provide more precise and detailed information on postural quality than mere duration of a standing task such as in the BOT-2 and in the 3 timed tasks used. Indeed, instrumenting subjects (i.e., using inertial sensors with accelerometers) was shown to be more sensitive than the static standing balance tests to find postural differences between mTBI and matched healthy control subjects (55).

### Clinical Implications

The present results reveal the importance of dynamic visual processing when testing postural stability after mTBI. It is suggested that VR technology can help to design more ecological and challenging tasks to assess postural stability compared to standard clinical tests. However, a limitation of our study is the lack of knowledge of the impact of these laboratory results on functional postural stability in everyday life and in physical activities. Postural problems last longer than the typical symptoms which are either no longer significantly reported nor related with postural deficits 3 months post-injury. This raises three important questions.

First, knowing that absence of symptoms is still one of the main factors determining return to play in active sports (56), and knowing that postural deficits can be found much later than disappearance of reported symptoms: are we returning children too early to sports and physical activities requiring postural control? In this respect, we must consider that there is a strong increased probability of sustaining a second TBI after a first one (57), and the odds of sustaining an acute lower extremity musculoskeletal injury during a 90-day period after return to play could be 2.48 times higher in concussed athletes compared to controls during the same period (58). More and more evidence suggest that athletes are more likely (odds 1.5-3) to suffer a lower extremity musculoskeletal injury in the year following their concussion, compared to control participants (25–27, 42, 59, 60). Deficits in processing dynamic visual inputs could result in slower reaction time when a motor response is required and thus could threaten not only performance, but also the postural stability and security of the child. It has been recently suggested that perceptual-motor control may be implicated in the increased risk of musculoskeletal injury (61). The authors suggested that the disruption of the perception-action coupling caused by mTBI (more precisely, by symptoms such as fatigue or ocular dysfunction) would increase the risk of subsequent injury due to the improper temporal execution of movements and/or incorrect body positioning in response to affordances of the environment. They proposed that the perception-action coupling loop should be re-established through post-concussion rehabilitation to avoid the risk of subsequent musculoskeletal injury (61). In that matter, the authors have proposed a computerized perception-action coupling task that seems promising to complement current concussion assessments (61, 62).

Second, since duration of postural deficits last longer than 3 months, but appear to recover by 12 months, could physical rehabilitation, aiming at improving sensorimotor integration (including visual, proprioceptive and vestibular) during complex dynamic postural tasks, shorten the duration of the recovery? Obviously, future studies evaluating rehabilitation protocols are needed in children with mTBI (63–65). There have been indications that rehabilitation focusing on postural training can improve postural control in adults with severe TBI (66–68) and that such an improvement is accompanied by alterations in cerebellar white matter (69). Nevertheless, more research employing higher quality methodological designs and challenging VR environments are clearly needed to better assess the efficacy of current acquired brain injury rehabilitation strategies (48, 70–72).

Third, are we sufficiently testing the complex aspects of sensorimotor integration involved in postural control? In addition to motor response to visual inputs, we emphasize the importance of testing dynamic postural stability as well as static postural stability (73). Present recommendations and assessment tools such as the Child-SCAT5 for children aged 5 to 12 years (56, 74, 75) do include postural examination items such as the double leg stance and tandem stance eyes closed (static conditions), but have removed tandem gait on ground (a dynamic balance condition). Since Gagnon et al. (9) found that differences between children with mTBI and healthy controls were most apparent during more challenging dynamic conditions such as tandem gait on a balance beam and stepping over an obstacle, we suggest that such tests should be added to assess dynamic visuomotor responses after mTBI in children. Moreover, the development of advanced technology measuring vRMS and BSA, such as the Visual Tunnel Paradigm, could help to detect subtle postural impairments and, eventually, guide rehabilitation.

### Limitations

There are some limitations associated with the results of the present study. First, it will be important to replicate the results of this study with larger sample sizes. In addition, considering the duration of the testing window (1 year), it will be important to monitor factors such as the level of physical activity during the full testing duration as this could have implication for postural stability in children (76, 77). It also must be noted that the scale used for post-concussion symptom assessment was the one included in the ImPACT test, which has been normed for children as of age 11 but there are 3 children aged 9 and 10 years in each group, for whom scores could be less valid. Regarding the VR postural test, it will be of interest to validate whether measurements taken from the lower back (closest to the center of mass), which have shown very good accuracy (98.42%) for fall detection, provide similar or more accurate results of postural response compared to measures taken from the head position (78), which have shown good accuracy (96.61%) for fall detection in a previous study (79).

## Conclusion

This study revealed that children with mTBI presented with postural anomalies when compared to uninjured children and that these anomalies were still present 3 months post-mTBI, but not at 12 months. Postural deficits were not salient in traditional clinical measures of balance (BOT-2; timed tasks), nor systematically predicted by reported post-concussion symptoms magnitude. This preliminary study suggests that controlled dynamic postural measures using a VR environment and involving processing of 3D visual inputs, which mimic ecological optic flow, could help guide the development of a sensitive clinical tool to detect subtle postural impairments and, eventually, guide rehabilitation.

## Data Availability Statement

The raw data supporting the conclusions of this article will be made available by the authors, without undue reservation.

## Ethics Statement

The studies involving human participants were reviewed and approved by the Research Ethics Board of the Université de Montréal and the Research Ethics Board of the McGill University Health Centre. Written informed consent to participate in this study was provided by the participants' legal guardian/next of kin.

## Author Contributions

RF, MM, JF, and IG conceived, designed, and supported this research. RA programmed the Virtuel tunnel paradigm. TR and SG conducted the present study and collected the primary data. TR, RF, SG, and IG drafted the manuscript. TR, IG, RA, MM, and JF revised the manuscript. All authors of this work met ICMJE criteria for authorship, made substantial contributions to the conception and design, acquisition of data, analysis and interpretation of data, drafting, critical revising, and final approval of this manuscript.

## Funding

This project was supported by the Canadian Institutes of Health Research (grant # MOP-18004).

## Conflict of Interest

The authors declare that the research was conducted in the absence of any commercial or financial relationships that could be construed as a potential conflict of interest.

## Publisher's Note

All claims expressed in this article are solely those of the authors and do not necessarily represent those of their affiliated organizations, or those of the publisher, the editors and the reviewers. Any product that may be evaluated in this article, or claim that may be made by its manufacturer, is not guaranteed or endorsed by the publisher.
